# An all-optical technique enables instantaneous single-shot demodulation of images at high frequency

**DOI:** 10.1038/s41467-019-14142-w

**Published:** 2020-01-28

**Authors:** Swapnesh Panigrahi, Julien Fade, Romain Agaisse, Hema Ramachandran, Mehdi Alouini

**Affiliations:** 1 Univ Rennes, CNRS, Institut FOTON-UMR 6082, F-35000 Rennes, France; 2 Raman Research Institute, Sadashiv Nagar, Bangalore, Karnataka 560080 India

**Keywords:** Electrical and electronic engineering, Imaging and sensing

## Abstract

High-frequency demodulation of wide area optical signals in a snapshot manner remains a technological challenge. If solved, it could open tremendous perspectives in 3D imaging, vibrometry, free-space communications, automated vision, or ballistic photon imaging in scattering media with numerous applications in smart autonomous vehicles and medical diagnosis. We present here a snapshot quadrature demodulation imaging technique, capable of estimating the amplitude and phase from a single acquisition, without synchronization of emitter and receiver, and with the added capability of continuous frequency tuning. This all-optical optimized setup comprises an electro-optic crystal acting as a fast sinusoidal optical transmission gate, and allows four quadrature image channels to be recorded simultaneously with any conventional camera. We report the design, experimental validation and examples of applications of such wide-field quadrature demodulating system that allowed snapshot demodulation of images with good spatial resolution and continuous frequency selectivity up to a few 100s of kilohertz.

## Abstract (web summary)

Traditional lock-in detection methods have been limited for wide-field imaging. Here, the authors present an all-optical design which enables four quadrature image channels to be recorded simultaneously, and show demodulation of wide-field images based on a single frame acquisition.

## Introduction

Lock-in detection is an ubiquitous measurement technique, where the signal of interest is imparted a periodic variation at the source to distinguish it from the random noise that it acquires on its path to the detector. Extremely weak signals may thus be extracted by selectively amplifying, at the detector, the component at the modulation frequency. In the field of optics, light intensity modulation/demodulation techniques have been employed, for example, in telemetry, free-space communications, biomedical imaging and viewing through scattering media. The enhanced immunity to noise and the massively increased transmission bandwidth due to multiplexed modulation has been widely used in telemetry. On the other hand, a weakly modulated signal may be intentionally cloaked in noise, with several different messages being encoded at different frequencies. Despite the eventuality of an eavesdropper demodulating a message at a certain frequency, the presence of multiple, possibly contradictory messages at different frequencies still provides partial secrecy (or at least discretion) of the communication.

Another very important area where the modulation/demodulation technique plays a dominant role is in the imaging through complex disordered media. Optical inhomogeneities within the medium indeed cause random multiple scattering of photons, altering the normally ballistic transport of light into diffusive transport, which strongly degrades the image-bearing capabilities of the light, hence resulting in turbidity, and poor visibility, as in the case of biological tissues or in fog. Imaging of objects hidden in such media can be achieved by the extraction of the ballistic photons, that constitute a very small fraction of the total photons reaching the detector, but which retain the information of the source (direction, state of polarization, spatial and temporal modulation) and can lead to direct imaging through a turbid medium. On the other hand, indirect imaging of embedded objects can be performed by estimation of optical inhomogeneities in the turbid medium from the detected scattered photons. As a result, imaging through complex disordered media has been addressed using various techniques ranging from the efficient but costly time-gated techniques^1,2^, to the comparatively inexpensive polarization imaging^3–5^ and spatial modulation techniques^6^. In this context, the temporal modulation/demodulation technique utilizes the fact that the forward scattered ballistic light travels in a straight-line path within the medium, maintaining a phase relationship with the modulation of the source, while the scattered diffusive light has a statistical distribution of paths and hence loses the unique phase relation with the source, allowing its contribution to be filtered out for sufficiently high modulation frequencies. For instance, modulation frequencies in the range of 10–100 MHz would meet such requirement for transport applications (or for usual 3D range-imaging applications), whereas imaging in biological scattering tissues would require very high frequency operation in the gigahertz domain^7^. As a result, modulation-based approaches in the radio-frequency (RF) range have been confined so far to point-wise detection configurations^8,9^. Regarding two-dimensional ballistic-light imaging, existing techniques invariably require some form of processing at the receiver, either electronically, mechanically, or via software, increasing the complexity of the system, and often, the processing time. For example, electronic lock-in detection permits demodulation at one location at a time, necessitating a step-scan of the detector^4^. Software based approaches obviate the need for a step scan, but the requirement of obtaining images in real-time restricts the length of the time-series that may be recorded for demodulation, thus limiting the frequency of use^3,10^ well below the 100 MHz–GHz range.

Clearly, rapid techniques providing wide-field demodulation imaging are greatly desirable as they would not only permit real-time applications like navigation, but would also open up possibilities for 3D ranging and imaging, vibrometry, optical communications, and specialized scientific instrumentation. Such imaging at high frequencies would be a leap forward for imaging through turbid media, a field of interest that has bearing on vision through opaque scattering walls^10–15^, medical diagnosis^16–19^, food quality analysis^20^, transport safety^5,21,22^, underwater vision^23^ and imaging through fog^3^. Progressing from single-pixel lock-in detection to simultaneous demodulation over millions of pixels to achieve snapshot image demodulation would bring to the realm the observation of spatially distributed and fast physical effects that remain otherwise undetectable. However, demodulation of light at radio frequencies and higher is known to present several practical challenges like phase synchronization, timing jitters, snapshot operation and difficulty in frequency tuning that have only been partially addressed by the few existing laboratory demonstrations of full-field demodulation, based on image intensifiers, Time-of-Flight (ToF) sensors, lidar systems^24–28^. While fast intensity-modulated light sources are easily available, full-field demodulation of images at high frequencies still awaits a viable solution. This calls for a radically new approach to modulation/demodulation imaging in order to overcome technological impediments to rapid, full-field imaging.

In this article, we propose and demonstrate an imaging technique where the demodulation at the receiver is performed optically to obtain two-dimensional images instantaneously from a recording of a single frame of an ordinary digital camera. We first report the principle of this Full-field All-optical Single-shot Technique for Quadrature Demodulation (FAST-QUAD) which is in principle compatible with high-frequency operation up to the RF range. Then, the experimental validation of this imaging concept is provided using a first prototype which is described and characterized. We demonstrate demodulation imaging with good resolution (300 × 300 pixels) on the estimated amplitude and phase images in the DC to 500 kHz frequency range, with continuous frequency tuning capability, and without synchronization between source and observer. We finally illustrate the interest and versatility of this technique on two practical scenarii of use, showing that it would be equally applicable to spatially-multiplexed free-space communications, cryptography and ballistic light imaging with potential high impact on numerous applications such as smart autonomous vehicles technologies and medical diagnosis.

## Results

### Full-field all-optical singleshot technique for quadrature demodulation

Full-field instantaneous single-shot demodulation of images imaging can be achieved by performing the demodulation of the intensity-modulated light source(s) in the polarization-space at the receiver. For that purpose, we exploit the Pockel’s effect in an electro-optic crystal that introduces a phase difference between two orthogonal components of light that is proportional to an applied voltage. This effect occurs at very high speeds, with response times of a few picoseconds^29^. As detailed below, suitable electrical excitation of the electro-optic crystal and orientation of birefringent/polarizing optical elements automatically resolves light into the quadrature components, and achieves the demodulation obviating the need for phase synchronization with the source, the only requirement to achieve snapshot demodulation being that the FAST-QUAD device is tuned to the frequency of the intensity modulation. Final image integration on a standard camera is performed during an exposure time that is several orders of magnitude larger than the modulation period, and thus a single frame captured by the FAST-QUAD camera provides the demodulated full-field image.

**Fig. 1:**
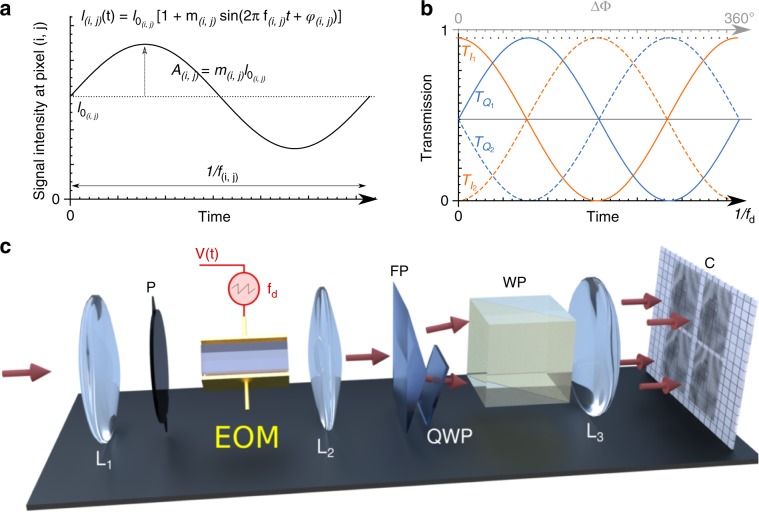
Principle of quadrature demodulation imaging and FAST-QUAD concept. **a** At a given location (pixel) $(i,\,j)$ of the scene, the input light is assumed to be intensity-modulated at frequency ${f}_{(i,j)}$ with modulation index ${m}_{(i,j)}$, over a mean (DC) intensity component ${I}_{{0}_{(i,j)}}$. **b** At each pixel of the detector, the incoming light is demodulated at frequency ${f}_{{\rm{d}}}$ by product demodulation through four transmission-modulated optical gates along four quadratures (${T}_{{I}_{1}}$, ${T}_{{Q}_{1}}$, ${T}_{{I}_{2}}$, ${T}_{{Q}_{2}}$). It is then time-averaged on the camera over the exposure time of a frame, and the four intensity values (${I}_{{1}_{(i,j)}}$, ${Q}_{{1}_{(i,j)}}$, ${I}_{{2}_{(i,j)}}$, ${Q}_{{2}_{(i,j)}}$) obtained at each pixel allow the average (DC) intensity ${I}_{{0}_{(i,j)}}$, amplitude ${A}_{(i,j)}={m}_{(i,j)}\ {I}_{{0}_{(i,j)}}$ and phase ${\varphi }_{(i,j)}$ of light modulated at frequency ${f}_{{\rm{d}}}$ to be simultaneously retrieved over the entire scene. **c** The FAST-QUAD technique consists of the simultaneous recording of four sub-images, on a single camera frame, each of which is the result of product demodulation, at frequency ${f}_{{\rm{d}}}$, of the incoming light with specific phases (${0}^{\circ }$, $9{0}^{\circ }$, $18{0}^{\circ }$, and $27{0}^{\circ }$). From these four sub-images, the average (DC) intensity and the modulation amplitude and phase maps can be obtained in a snapshot manner from a single acquired frame. To achieve this instantaneously and optically, the input light/image is passed through a lens ${L}_{1}$, a polarizer (P), and an electro-optic (EO) crystal driven by a high-voltage sawtooth signal at frequency ${f}_{{\rm{d}}}$, which results in an optical phase difference excursion over $36{0}^{\circ }$ between two orthogonal polarization components of light. After collimation through lens ${L}_{2}$, the beam is split by a Fresnel bi-prism (FP), with the lower beam undergoing an additional $9{0}^{\circ }$ optical phase shift passing through a quarter-wave plate (QWP). A polarizing Wollaston prism (WP) and a lens ${L}_{3}$ complete this 4-channel voltage-controlled sinusoidally varying optical transmission gate.

Indeed, contrary to standard approaches that rely on temporal sampling of modulated intensity signals (through image intensifiers, or specific electronic chips such as ToF sensors), FAST-QUAD requires no discrete temporal sampling of the received data. Also, contrary to anterior work using temporally multiplexed quadrature demodulation in optical coherence tomography (OCT)^30,31^, FAST-QUAD performs lock-in demodulation continuously in time and simultaneously over the full spatial extent of the image. This is achieved by transferring the well-known quadrature demodulation (lock-in) principle to the optical domain and in a massively spatially-multiplexed way in order to handle image demodulation. A classic electronic lock-in detector multiplies an incoming signal that is modulated at a frequency $f$ by a sinusoid at frequency ${f}_{{\rm{d}}}$ generated by a local oscillator. The frequency and phase of this oscillator is tuned to obtain a coherent match with the weak incoming signal of interest. The product of the two is integrated over a length of time to average out all components except the one of interest. The phase matching step can be avoided when quadrature demodulation is performed, i.e., when the signal is demodulated by two local oscillators in quadrature (i.e., with a $9{0}^{\circ }$ phase delay between each other) to obtain two demodulation channels ($I$ and $Q$ quadratures). Optically, the mathematical operation of multiplication of an incoming intensity-modulated light signal (or image) at a frequency $f$ with a local oscillator at ${f}_{{\rm{d}}}$ can be achieved by passing the input light (or image) through an optical gate whose transmittivity is modulated sinusoidally at frequency ${f}_{{\rm{d}}}$. Contrary to electronics, optical quadrature lock-in detection requires 4 transmission gates (${T}_{{I}_{j}},{T}_{{Q}_{j}},j=1,2$) oscillating at ${f}_{{\rm{d}}}$, with phases separated by $9{0}^{\circ }$ because the incoming light passes through optical transmission gates with non-zero transmittivity mean. This is shown schematically in Fig. 1 for transmissions (${T}_{{I}_{1}}=(1+\cos2\pi {f}_{{\rm{d}}}t)/2$, ${T}_{{Q}_{1}}=(1+\sin 2\pi {f}_{{\rm{d}}}t)/2$, ${T}_{{I}_{2}}=(1-\cos2\pi {f}_{{\rm{d}}}t)/2$, ${T}_{{Q}_{2}}=(1-\sin 2\pi {f}_{{\rm{d}}}t)/2$, as represented in Fig. 1b), these latter corresponding to four quadratures with respective phases 0, 90, 180, and 270 degrees.

Such parallel and instantaneous demodulation is achieved by use of a suitably designed arrangement of birefringent elements at the input of a standard low frame-rate camera (CCD or CMOS). The specific optical architecture employed for this purpose is illustrated in Fig. 1c. It comprises a polarizer (P), a quarter-wave plate (QWP) and splitting/polarizing prisms (FP, WP), and a single electro-optic (EO) crystal (e.g., Lithium Niobate (LiNbO${}_{3}$)) with eigenaxes oriented at $4{5}^{\circ }$ from the input polarization axis imposed by the polarizer P. A periodic sawtooth electric field at frequency ${f}_{{\rm{d}}}$, with sufficient excursion to ensure perfect sinusoidal optical transmission, (i.e., allowing a $36{0}^{\circ }$ excursion of the optical phase difference) is applied to the EO crystal. As a result, this architecture does not require any dephasing or splitting electronic circuit. The $\lambda /4$ optical path difference arising in the QWP is advantageously converted into a $9{0}^{\circ }$ phase delay between optical transmission curves ${T}_{{I}_{j}}$ and ${T}_{{Q}_{j}}$, whatever be the demodulation frequency ${f}_{{\rm{d}}}$, thereby offering huge tuning capabilities of the demodulation imaging setup. The integration stage employed in conventional electronic quadrature lock-in demodulation circuits is here simply and directly performed by acquiring a single frame on the standard camera (C) for a typical duration of several thousand modulation periods. The optical arrangement is so designed that it allows the four quadrature images (${I}_{1}$, ${Q}_{1}$, ${I}_{2}$, ${Q}_{2}$) to be simultaneously acquired on the camera (see Fig. 1c).

Finally, at each pixel $(i,\,j)$ of the scene, the average (DC) intensity ${I}_{{0}_{(i,j)}}$, as well as the amplitude ${A}_{(i,j)}$ and phase ${\varphi }_{(i,j)}$ of the light component modulated at frequency ${f}_{{\rm{d}}}$ can be retrieved from the four detected intensities, since

1a $${I}_{{0}_{(i,j)}}=\frac{{I}_{{1}_{(i,j)}}+{I}_{{2}_{(i,j)}}+{Q}_{{1}_{(i,j)}}+{Q}_{{2}_{(i,j)}}}{4},$$

1b $${A}_{(i,j)}=\sqrt{{({I}_{{1}_{(i,j)}}-{I}_{{2}_{(i,j)}})}^{2}+{({Q}_{{1}_{(i,j)}}-{Q}_{{2}_{(i,j)}})}^{2}},$$

1c $${\varphi }_{(i,j)}={\rm{atan}}\left[\frac{{Q}_{{1}_{(i,j)}}-{Q}_{{2}_{(i,j)}}}{{I}_{{1}_{(i,j)}}-{I}_{{2}_{(i,j)}}}\right],$$

hence allowing instantaneous quadrature demodulation over the entire image from a single acquired frame.

### Experimental demonstration

A prototype of a FAST-QUAD camera which implements the optical setup of Fig. 1c has been designed and built in order to validate and demonstrate the potentialities of the proposed full-field quadrature demodulation imaging approach. It includes a $40\times 2\times 2$ mm${}^{3}$ lithium niobate (LiNbO${}_{3}$) EO crystal and a low-frame rate high-dynamic range camera (Andor NEO sCMOS, 5.5 Mpixels, 16 bits) and was able to be operated up to few hundreds of kilohertz so far, limited by the bandwidth of the high-voltage amplifier available in our laboratory. The technical considerations and design of the prototype are reported in Methods. The data processing pipeline and calibration procedure developped to compensate for the mechanical and optical imperfections of this first prototype are given in Supplementary Notes 1 and 2, and Supplementary Figs. 1 and 2.

The experimental validation reported below was performed with an externally intensity-modulated green laser illumination ($\lambda =532$ nm), to limit the effect of the strong chromatic dispersion occuring in the optical components (especially EO crystal, prisms and QWP). In future developments and applications of FAST-QUAD, direct modulation of the LEDs or laser light sources can be envisaged. A complete description of the imaging scenes considered in the remainder of this article is given in Supplementary Note 3 and Supplementary Fig. 3.

**Fig. 2:**
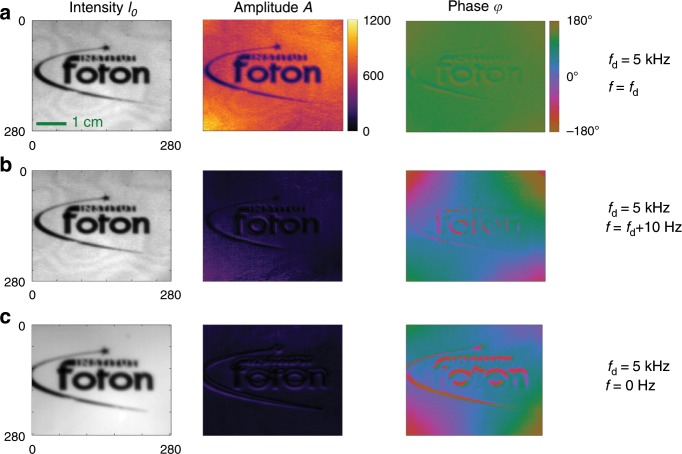
Demonstration of snapshot image demodulation with FAST-QUAD. In this grid of images, the left column contains the mean (DC) intensity of the source (the logo of Institut FOTON), the central column contains the amplitude map and the right column the phase map obtained by demodulation at frequency ${f}_{d}$ by FAST-QUAD. **a** The first row corresponds to the case where the scene is uniformly illuminated with laser light modulated at $f={f}_{{\rm{d}}}$, thereby resulting in strong demodulated amplitude signal and uniform phase map. **b** The second row corresponds to the source being modulated at $f={f}_{{\rm{d}}}+10$ Hz, resulting in almost null demodulated amplitude. **c** The third row corresponds to the case where the source is unmodulated, hence allowing to mimic illumination by spurious ambient light. In this case, the demodulated amplitude is negligible too.

Instantaneous full-field demodulation imaging using FAST-QUAD has first been validated on a simple imaging scenario where the source comprised a logo of the Institut Foton (see Fig. 2) that was homogeneously illuminated by light modulated at $f=5$ kHz.

The demodulation frequency on FAST-QUAD was first tuned to the exact modulation frequency by setting ${f}_{{\rm{d}}}=5$ kHz, and the corresponding results are shown in Fig. 2a. The average (DC) intensity map of the incoming signal, the estimated amplitude map and the estimated phase map after demodulation are displayed. In this case, the modulation amplitude is well retrieved, with the amplitude map showing a good homogeneity throughout the field of view (FOV) ($280\times 280$ pixels), and an appreciable spatial resolution. As expected, the demodulated phase leads to a fairly flat estimated phase map. The quality of the demodulated images demonstrates the efficiency of the calibration/processing algorithms developed to compensate for the imperfections of the optical setup.

The demodulation frequency was then slightly detuned by setting $\Delta f=f-{f}_{{\rm{d}}}=10$ Hz, while maintaining the illumination intensity as illustrated in Fig. 2b. In this case, the demodulated amplitude map in Fig. 2b is almost dark, showing only a very low-contrast residual image of the logo. The phase map is of course not flat anymore, and would be expected to be irrelevant when the receiver is not tuned with the emitter’s frequency. The smooth estimated phase pattern observed here is due to a residual lack of correction of the phase mismatch across the isogyre pattern (see Methods and Supplementary Note 1) that spreads across the raw images and which is corrected by calibration and post-processing. It was also checked that the demodulated amplitude was negligible when the scene was illuminated by unmodulated white light, as shown in Fig. 2c, to simulate strong ambient illumination. These first results obtained at $f=5$ kHz hence demonstrate the ability of the FAST-QUAD approach to efficiently demodulate an image in a snapshot manner with a good image quality and resolution.

**Fig. 3:**
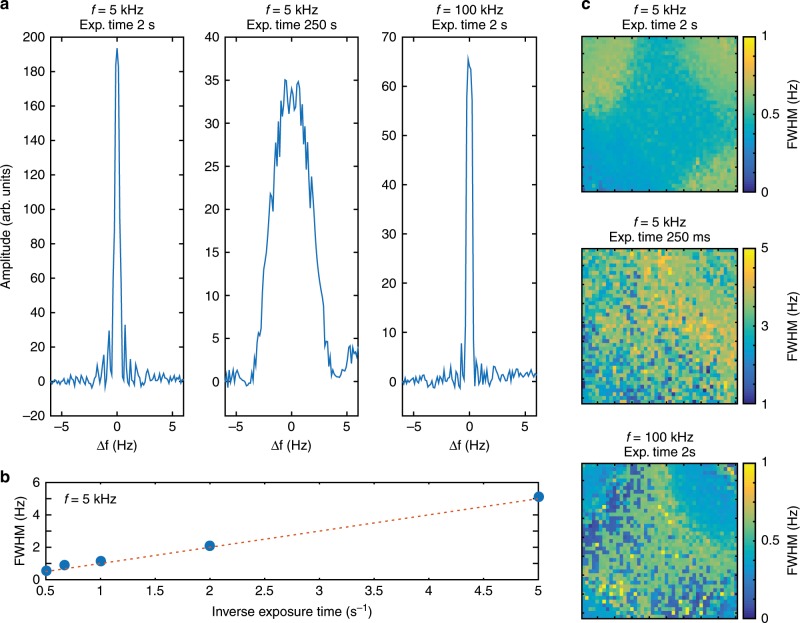
Evaluation of frequency selectivity. **a** Demodulated amplitude averaged over $300\times 300$ pixels plotted as a function of frequency detuning $\Delta f=f-{f}_{{\rm{d}}}$ with steps of 0.12 Hz for $f=5$ kHz and 2 s exposure time (left); $f=5$ kHz and 250 ms exposure time (center); $f=100$ kHz and 2 s exposure time (right). **b** FWHM of the demodulation efficiency scales as the inverse exposure time. **c** Uniformity maps of the frequency selectivity (FWHM of the demodulated intensity as a function of $\Delta f$) over the entire FOV ($8\times 8$ pixels binning).

The demodulation frequency of FAST-QUAD can be continuously and easily tuned. To explore the possibility of utilizing this property to distinguish between images modulated at closely separated frequencies, we investigated the frequency selectivity of FAST-QUAD on a homogeneous scene. The average demodulated amplitude across the FOV was evaluated as a function of the frequency detuning $\Delta f$. A demodulation bandwidth of ~0.5 Hz (evaluated at full width half maximuim (FWHM)) is obtained at $f=5$ kHz and $f=100$ kHz for a 2-s exposure time on the FAST-QUAD camera (Fig. 3a); this increases to ~2 Hz when the exposure time is reduced to 0.5 s. Similar to usual lock-in detection setups, the scaling of the selectivity with exposure time is an expected result which is confirmed by Fig. 3b, where the FWHM of the demodulated amplitude is plotted as a function of the exposure time for $f=5$ kHz. Next, the uniformity of the frequency selectivity across the FOV of the camera is analyzed in Fig. 3c, where the spatial evolution of the FWHM of the demodulated amplitude is displayed using an $8\times 8$ pixels binning. The selectivity of FAST-QUAD is found to be quite uniform across the FOV, although the homogeneity is degraded with this first prototype when the global demodulation efficiency decreases with higher modulation frequency or reduced exposure time. Experimentally, it was observed that 500 kHz corresponded to the frequency cut-off after which no significant demodulation efficiency could be obtained with this first prototype, due to the limited bandwidth of the high-frequency voltage amplifier used.

## Discussion

The previous section has shown the real-time image demodulation of a single source modulated at a single frequency. We now illustrate two important potential applications of FAST-QUAD: the reduction of clutter and image encryption.

**Fig. 4:**
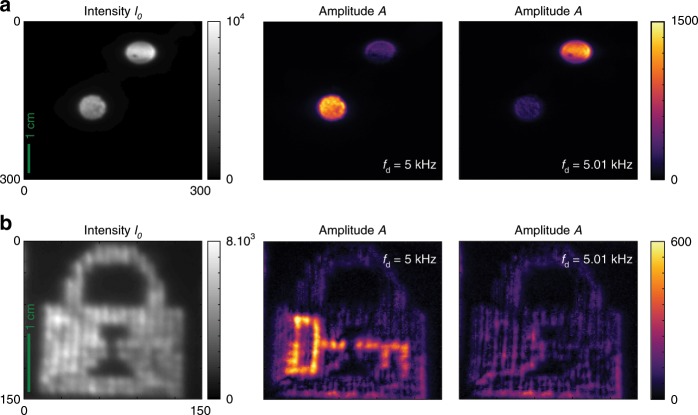
Experimental illustration of potential applications of FAST-QUAD. **a** Demonstration of frequency tuning: two laser spots of equal intensity and equal modulation index, but of distinct modulation frequencies (respectively 5.00 kHz and 5.01 kHz) are imaged and demodulated with FAST-QUAD (exposure time 2 s). Left: Estimated average intensity map when demodulation frequency is set to ${f}_{{\rm{d}}}=5$ kHz or ${f}_{{\rm{d}}}=5.01$ kHz. Right: Modulation amplitude maps demonstrate that FAST-QUAD can be continuously tuned to any demodulation frequency, making it possible to actively select and discriminate emitters at different frequencies. **b** Demonstration of image encryption with modulated light: an intensity-modulated object (key) is concealed in an unmodulated background (lock), and the scene is imaged with FAST-QUAD (exposure time 1 s). Left: The encrypted image cannot be detected on a conventional intensity camera. Right: Modulation amplitude maps demonstrate that FAST-QUAD can retrieve the concealed image when ${f}_{{\rm{d}}}$ is set to the exact frequency used by the sender.

The imaging experiment presented in Fig. 4a makes use of the continuous frequency discrimination capability of FAST-QUAD. Two objects (in our case, two disks) are illuminated by two independent intensity modulated sources with the same average intensity (see Fig. 4a) but slightly different modulation frequencies (5.00 kHz and 5.01 kHz). Tuning the demodulation frequency ${f}_{{\rm{d}}}$ to one or the other frequency immediately results in a snapshot filtered image of the object modulated at that frequency in the amplitude map, demonstrating the frequency selectivity of FAST-QUAD (Fig. 4a). Such discrimination capability opens up the possibility of using several sources at different modulation frequencies, permitting novel imaging applications like assigning distinct frequencies to different classes of emitters, (e.g., vehicles, road signs, landing areas, etc.), and viewing each class in a de-cluttered fashion, by demodulating at their specific modulation frequency. As it requires no synchronization between the source and the receiver, the technique can be employed in the presence of relative motion between the source and receiver. This could, for example, help de-clutter the view of a pilot as he approaches for landing, and could aid in road, rail and other forms of navigation.

The second experiment, presented in Fig. 4b, utilizes the fact that FAST-QUAD provides frequency selectivity, and also requires no synchronization between the source and the receiver. It illustrates how a piece of secret information or an image (here, a picture of a key) could be embedded in decoy background (here, a picture of a lock) by the sender, and retrieved by the intended recipient equipped with FAST-QUAD, whereas it would go totally unnoticed by any other observer using a conventional camera. As the hidden object is intensity-modulated at high frequency (here, 5 kHz) but with same average intensity as the unmodulated background, a conventional camera does not allow for its detection (see intensity map in Fig. 4b). On the other hand, a FAST-QUAD camera with the same exposure time makes it possible to detect the encrypted image efficiently (see amplitude maps in Fig. 4b), when ${f}_{{\rm{d}}}$ is set to the frequency used by the sender, without need for phase synchronization. A mismatch in the frequencies fails to reveal the embedded image (Fig. 4b).

In conclusion, we have proposed and demonstrated a novel all-optical technique for instantaneous full-field demodulation of images from a single recorded frame of an ordinary digital camera. By means of illustrative examples, we demonstrate the potential of this approach which has numerous additional advantages like requiring no synchronization between source and observer (thereby permitting relative motion), continuous frequency tuning capability, compactness and portability. To the best of our knowledge, this proof-of-principle experiment is the first ever snapshot demodulation imaging in the DC to 500 kHz frequency range, with the added capability of continuous frequency tuning.

These first encouraging results act as an incentive for achieving high-frequency snapshot quadrature demodulation imaging in the 10’s MHz to 10’s GHz range, which is no longer unrealistic. This concept would bring a real breakthrough in the field of imaging in terms of applications, among which we can cite high-speed snapshot vibrometry, multiplexed free-space optical communications, automated vision, or spatially resolved lock-in detection at high-frequency for enhanced detection of spatially distributed physical phenomena, but also 3D imaging (potentially with range resolution of one millimeter or even below using modulated illumination in the ultra high frequency (UHF) range and above). More importantly, this would also enable ballistic photons imaging through scattering media, with major applications in smart autonomous vehicles technologies, transportation safety and in biomedical diagnosis for in-depth imaging in highly diffusive biological tissues. Since the technique is based on the electro-optic effect (e.g., Pockel’s effect with typical response times on the picosecond scale^29^), the underlying concept of FAST-QUAD should remain valid even at those very high frequencies. Technological bottleneck to operation at high frequencies would primarily be the fast application of high voltage signals across the EO crystal. This calls for an optimized optical design to limit the size of the EO crystal used while ensuring good image quality. In such a design, achromatic optical elements should be favored, and a crucial optimization of the physical dimensions of the crystal should be found in order to not only improve the numerical aperture and the resolution of the system, but also to release the current constraint of narrow-band laser illumination. Another challenge will be the generation of high-frequency voltage ramps to allow for high-frequency operation. Lastly, conflicting requirements on the laser illumination linewidth imposed by good demodulation and speckle removal in the images will also be investigated in future developments.

## Methods

### Optical design of the FAST-QUAD prototype

**Fig. 5:**
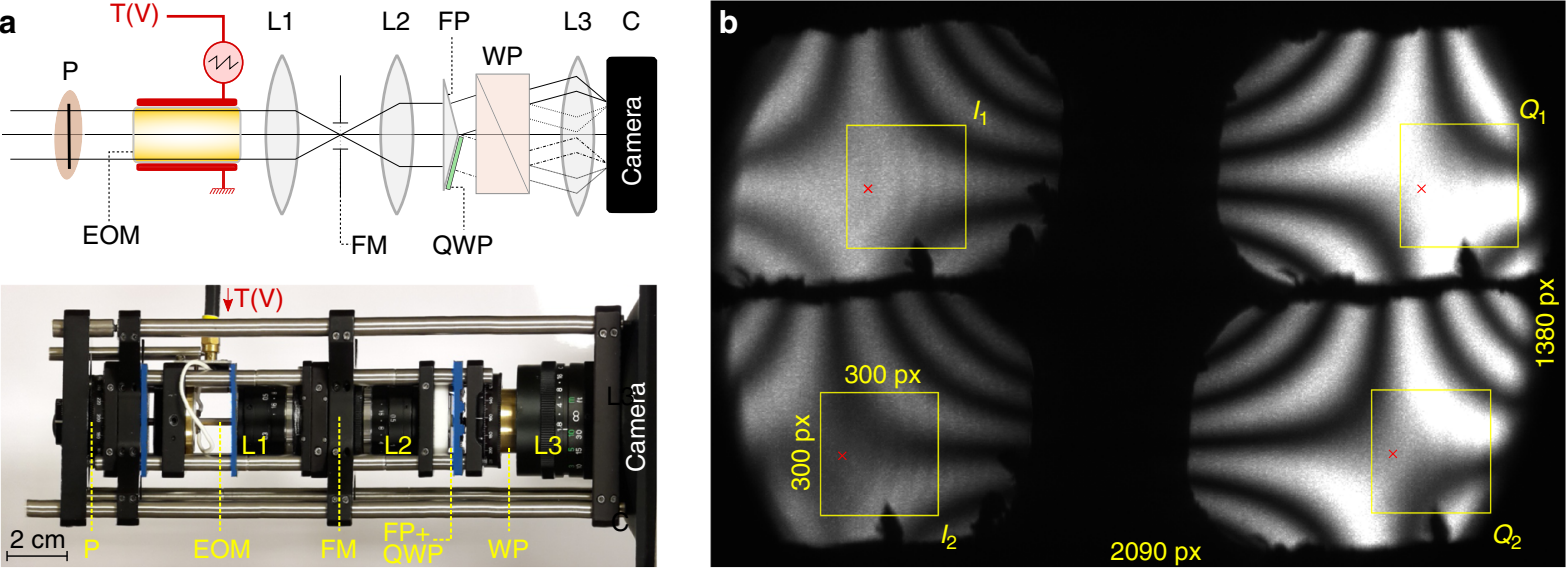
Presentation of FAST-QUAD prototype. **a** Schematic and photograph of the FAST-QUAD prototype. Due to the dimensions of the EO crystal employed ($2\times 2\times 40$ mm${}^{3}$), it was optimal to have it positioned after the input polarizer $P$. This was followed by a focusing lens ${L}_{1}$, a field mask (FM) at the intermediate image (that restricts the image spatial extent to prevent superimposition of the 4 sub-images on the camera), and thereafter a lens ${L}_{2}$ that recollimates the beam. A Fresnel biprism (FP) splits the beam into two, one part of which passes through a quarter-wave plate (QWP). Further propagation through a Wollaston prism results in 4 beams that are imaged onto the camera by means of lens ${L}_{3}$, providing the four quadrature images. **b** Example of raw image acquisition when the FAST-QUAD prototype is illuminated with homogeneous light field. The four $300\times 300$ pixels quadrature sub-images ${I}_{1}$, ${I}_{2}$, ${Q}_{1}$ and ${Q}_{2}$ are delineated with yellow frames. The red cross indicates the position of the reference pixel used in Supplementary Note 2 to illustrate the quadrature mismatch correction algorithm implemented.

The sketch and photograph of the optical setup used in this first prototype of FAST-QUAD are given in Fig. 5a. This prototype is based on the general design described in Fig. 1c, and as stated there, the central component of the prototype is an electro-optic (EO) crystal which introduces a controllable optical phase delay $\Delta \Phi (V)$ between two transverse components of the light beam propagated through it, when a voltage $V$ is applied across it. Assuming unpolarized incoming light with average intensity ${I}_{0}$, and an ideal input polarizer $P$ (see Fig. 1c), the light entering the EO crystal has intensity ${I}_{0}/2$ and is vertically polarized, i.e., its Jones vector reads ${{\bf{J}}}_{in}=\sqrt{{I}_{0}/2}\ {[1\quad 0]}^{T}$. The Jones matrix of the EO crystal whose eigenaxes are oriented at $4{5}^{\circ }$ from the input polarization reads

2 $$E{O}_{4{5}^{\circ }}\left[\Delta \Phi (V)\right]=\left[\begin{array}{ll}\cos\frac{\Delta \Phi (V)}{2}&i\sin \frac{\Delta \Phi (V)}{2}\\ i \sin \frac{\Delta \Phi (V)}{2}&\cos\frac{\Delta \Phi (V)}{2}\end{array}\right],$$

whereas the action of an ideal Wollaston prism (oriented along the input polarizer $P$) can be modeled with the following Jones matrices of a vertical and an horizontal polarizer:

3 $${{\rm{WP}}}_{H}=\frac{1}{\sqrt{2}}\left[\begin{array}{ll}1&0\\ 0&0\end{array}\right],\quad \,\text{and}\,\quad {{\rm{WP}}}_{V}=\frac{1}{\sqrt{2}}\left[\begin{array}{ll}0&0\\ 0&1\end{array}\right].$$

An ideal Fresnel bi-prism simply entails a splitting of the light intensity in half, with no effect on the beam polarization. Lastly, the QWP with its eigen-axes oriented at $-4{5}^{\circ }$ from the input polarization direction, has a Jones matrix equal to $QW{P}_{-4{5}^{\circ }}=E{O}_{4{5}^{\circ }}[-9{0}^{\circ }]$. As a result, classical Jones calculus following the path of the beams across the optical setup described in Fig. 5a leads to theoretical intensity transmission functions for the four quadrature channels:

4 $${T}_{{I}_{1}} 	= \, {\left|\frac{1}{2\sqrt{2}}{{\rm{WP}}}_{V}\cdot E{O}_{4{5}^{\circ }}\left[\Delta \Phi (V)\right]\cdot {\left[01\right]}^{T}\right|}^{2}\\ 	= \, \frac{1+\cos\Delta \Phi (V)}{8},\\ {T}_{{I}_{2}}	= \, {\left|\frac{1}{2\sqrt{2}}{{\rm{WP}}}_{H}\cdot E{O}_{4{5}^{\circ }}\left[\Delta \Phi (V)\right]\cdot {\left[01\right]}^{T}\right|}^{2}\\ 	= \, \frac{1-\cos\Delta \Phi (V)}{8},$$

and for the Q-quadratures:

5 $${T}_{{Q}_{1}}	= \, {\left|\frac{1}{2\sqrt{2}}{{\rm{WP}}}_{V}\cdot QW{P}_{-4{5}^{\circ }}\cdot E{O}_{4{5}^{\circ }}\left[\Delta \Phi (V)\right]\cdot {\left[01\right]}^{T}\right|}^{2}\\ 	= \, \frac{1+\sin\Delta \Phi (V)}{8},\\ {T}_{{Q}_{2}}	= \, {\left|\frac{1}{2\sqrt{2}}{{\rm{WP}}}_{H}\cdot QW{P}_{-4{5}^{\circ }}\cdot E{O}_{4{5}^{\circ }}\left[\Delta \Phi (V)\right]\cdot {\left[01\right]}^{T}\right|}^{2}\\ 	= \, \frac{1-\sin\Delta \Phi (V)}{8},$$

which, on varying V, provide the transmission curves described in Fig. 1b, thereby allowing spatially multiplexed lock-in product demodulation of the four quadratures.

To attain an optical phase delay $\Delta \Phi (V)$ modulated at the demodulation frequency ${f}_{{\rm{d}}}$ such that $\Delta \Phi (V)=2\pi {f}_{{\rm{d}}}t\ modulo\ 2\pi$, the voltage $V$ applied across the EO crystal must be linearly modulated along a sawtooth waveform with sufficient amplitude $V$. The well-known theory of EO crystals provides the relationship between $V$ and the optical phase difference. For instance, in the case of lithium niobate as used in this prototype, one has^32^ $\Delta \Phi (V)=\pi ({r}_{13}{n}_{O}^{3}-{r}_{33}{n}_{E}^{3})\ell /d\lambda \times V$, where ${r}_{13}\simeq 10$ pm.V${}^{-1}$ and ${r}_{33}\simeq 30$ pm.V${}^{-1}$ correspond to (Pockel’s) electro-optic coefficients of LiNbO${}_{3}$ (approximate values in the visible range)^33^. The ordinary, respectively, extraordinary, optical refractive indices of LiNbO${}_{3}$ are ${n}_{O}\simeq 2.32$ and ${n}_{E}\simeq 2.23$ at $\lambda =532$ nm^33^. To limit the voltage amplitude to reasonable values (i.e., within the ${100}-{200}$ V range), we selected a LiNbO${}_{3}$ EO crystal (Moltec GmbH) of length $\ell =40$ mm and with a $2\times 2$ mm${}^{2}$ aperture section ($d=2$ mm). A high-voltage sawtooth waveform (with peak-to-peak amplitude of 124 V calibrated to provide exact $36{0}^{\circ }$ maximum optical phase difference excursion $\Delta \Phi$) has been applied on the electrodes of the EO crystal using a signal generator (Tektronix AFG3252C) and a high-voltage amplifier (New Focus 3211 High Voltage Amplifier, ±$200$ V, 0–0.6 MHz bandwidth).

Due to the importance of the ratio of the length and thickness/breadth of the crystal, the best option in terms of FOV and resolution consisted of placing the EO crystal just after the input polarizer $P$ (Thorlabs, LPVISB050), before the image was focused by lens ${L}_{1}$ in an intermediate image plane where a field mask has been inserted to restrict the spatial extent of the image so that the 4 sub-images do not overlap at the camera (See Fig. 5a). The light is re-collimated through objective lens ${L}_{2}$ and split with a Fresnel bi-prism (NewLight photonics, $16{0}^{\circ }$ apex angle) and a Wollaston prism (Melles Griot, 15.9 mm, ${5}^{\circ }$ splitting angle). Lenses ${L}_{1}$, ${L}_{2}$ are two 25 mm focal length, $F/2.1$ camera objective lenses, whereas ${L}_{3}$ is a 50 mm, $F/2.8$ camera objective lens. As can be seen from Fig. 5a, the optical add-on offers relative compactness and ruggedness, which qualities could be further improved in future developments with an optimized optical and mechanical design.

A narrow bandwidth optical illumination was used with this setup (green laser illumination at $\lambda =532$ nm) to limit the detrimental effect on the image quality of the important chromatic dispersion occuring in optical components (especially EO crystal, prisms and QWP). As a consequence, the raw images acquired on the camera are spatially modulated across the FOV with an interference pattern known as isogyre, where the fringes correspond to light paths in the crystal of equal optical phase delays (birefringence)^29^. Such a pattern is clearly visible on the raw acquisition image example provided in Fig. 5b, but pixel-wise, the four interference patterns obtained on the camera were found to be advantageously in quadrature. As a result, homogeneous demodulated images were efficiently retrieved by implementing proper calibration and processing of frames, as reported in Supplementary Notes 1 and 2 and Supplementary Figs. 1 and 2.

## Supplementary information

Supplementary Information

## Data Availability

The datasets generated during and/or analyzed during this study are available from the corresponding author upon reasonable request.

## Code availability

The code used for data analysis, as well as for display of the data is available from the corresponding author upon reasonable request.
