# Supplementary material for: An all-optical technique enables instantaneous single-shot demodulation of images at high frequency
Source: Nat Commun. 2020 Jan 28;11:549. doi: 10.1038/s41467-019-14142-w (PMC6987108; doi:10.1038/s41467-019-14142-w)
Supplement: Supplementary file 1 — Supplementary Information [file 41467_2019_14142_MOESM1_ESM.pdf]

Supplementary Information

An all-optical technique enables instantaneous single-shot demodulation of images at high frequency

Panigrahi et al.

SUPPLEMENTARY NOTE 1: GENERAL DESCRIPTION OF THE CALIBRATION PROCEDURE AND PROCESSING PIPELINE

Optimal operation of the FAST-QUAD camera requires several calibration and processing steps to compensate for the mechanical and optical imperfections of the first prototype reported. The flowchart of the data processing involved to provide intensity, demodulated amplitude and phase maps from the raw image acquired is sketched in Supplementary Figure 1. Each processing step is based on calibration data obtained from preliminary experiments. Processing and calibration steps are briefly described here. Since FAST-QUAD relies on the snapshot acquisition of the four quadrature images on a single image sensor, a careful registration of these four sub-images is mandatory to provide final images with adequate resolution and to avoid estimation errors in the amplitude and phase maps. A calibration pattern is imaged with FAST-QUAD, allowing a first rough pixel-to-pixel registration to be performed, leading to the four ROIs depicted in Figure 5.b of the main article. However due to the optical aberrations that differ from one image channel to the other, it was necessary to implement a more precise image registration, involving: (a) estimation of the deformation maps during the calibration step on an appropriate calibration pattern (regular dotted grid); and (b) image interpolation of the I_2, Q_1, Q_2 images, so as to match the reference image I_1. Full details on the calibration and interpolation procedures implemented are not reported here for the sake of conciseness, as they are strictly similar to the ones described in Ref. [1], developed during prior work in the context of a two-channel polarimetric camera.

Mainly due to imperfect mechanical alignment of the optical components (polarizer, EO crystal, QWP, Wollaston prism), and to unbalanced optical throughput in the beam-splitting components (Fresnel biprism and Wollaston prism), the four sub-images retrieved from the raw data acquired on the image sensor may have differences in average intensity at each pixel, even in the case of perfect image geometrical registration. Such intensity mismatch is easily compensated for by applying appropriate correction maps on each of the four quadrature images, after which step the average intensity image can be directly estimated from the four quadrature images by $I_0 = \big(I_1 + I_2 + Q_1 + Q_2\big)/4$. The quadratures I_1 and I_2 (respectively, Q_1 and Q_2) have been checked to be in perfect opposite phase (180° phase shift) and to have balanced optical throughput. As a result, two quadrature signals $\tilde{I} = I_1 - I_2$ and $\tilde{Q} = Q_1 - Q_2$ are obtained by simple numerical subtraction. However, due to residual optical misalignments and experimental imperfections, the two signals retrieved appeared not to be perfectly in quadrature, which led us to implement a quadrature mismatch calibration/correction algorithm inspired from RADAR signal processing [2, 3] (The details of this quadrature mismatch calibration/correction algorithm is reported in Supplementary Note 2).

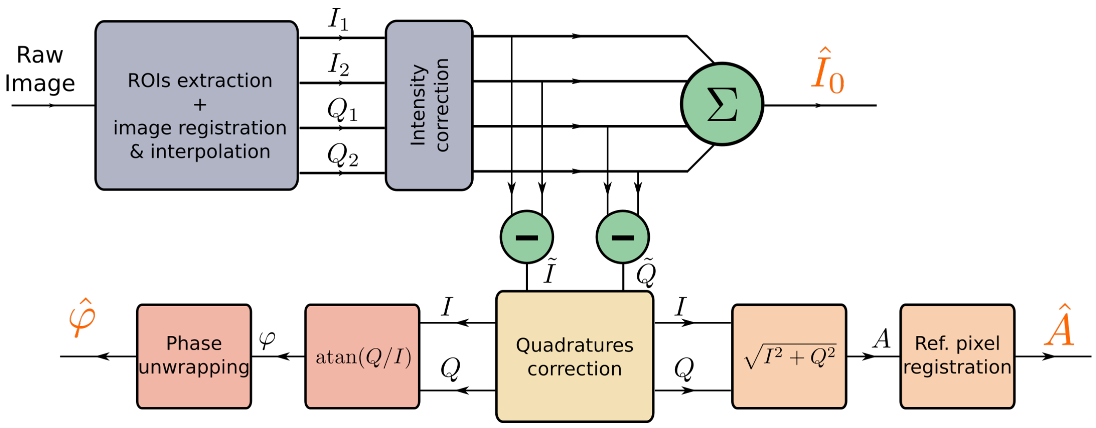

Supplementary Figure 1: Flowchart of the frames processing involved in FAST-QUAD. The correction procedure and the quadrature mismatch correction algorithm allow intensity $\hat{I}$, amplitude $\hat{A}$ and phase $\hat{\varphi}$ maps to be retrieved in a snapshot way from a single frame acquisition.

As sketched in Supplementary Figure 1, the corrected quadrature signals I and Q eventually allow the estimatd amplitude map $\hat{A}$ to be obtained after a final correction step, which is required to compensate for the possible spatial inhomogeneity of the demodulation efficiency across the FOV. Similarly, the estimated phase map $\hat{\varphi}$ is retrieved from the I and Q signals, after a phase unwrapping step to compensate for the inhomogeneous phase distribution imprinted by the isogyre pattern (see Methods). The inhomogeneity map and the phase unwrapping pattern are also calibrated from initial measurements on a homogeneous scene. The calibration procedure is not an easy task. Nevertheless, it has in principle to be performed once for a given imager. All experimental results presented in the manuscript have been obtained after a single, common, calibration procedure.

SUPPLEMENTARY NOTE 2: GENERAL DESCRIPTION OF THE CALIBRATION PROCEDURE AND PROCESSING PIPELINE

Due to experimental imperfections, the raw quadrature images obtained from subtraction of two image channels, respectively $\tilde{I} = I_1 - I_2$ and $\tilde{Q} = Q_1 - Q_2$, have small deviation from being in quadrature phase. Moreover, a residual amplitude mismatch was also observed between the two raw quadrature images $\tilde{I}$ and $\tilde{Q}$. Such differences are clearly visible on the raw quadrature data displayed in Supplementary Figure 2.a below, where the temporal evolution of the quadrature signals $\tilde{I}$ and $\tilde{Q}$ are plotted as a function of time for a reference pixel (denoted with symbol × in Figure 5 of the main article), while applying a slowly varying (0.1 Hz) voltage ramp on the EO crystal (with exposure time 350 ms, sampling period 400 ms on an homogeneous scene).

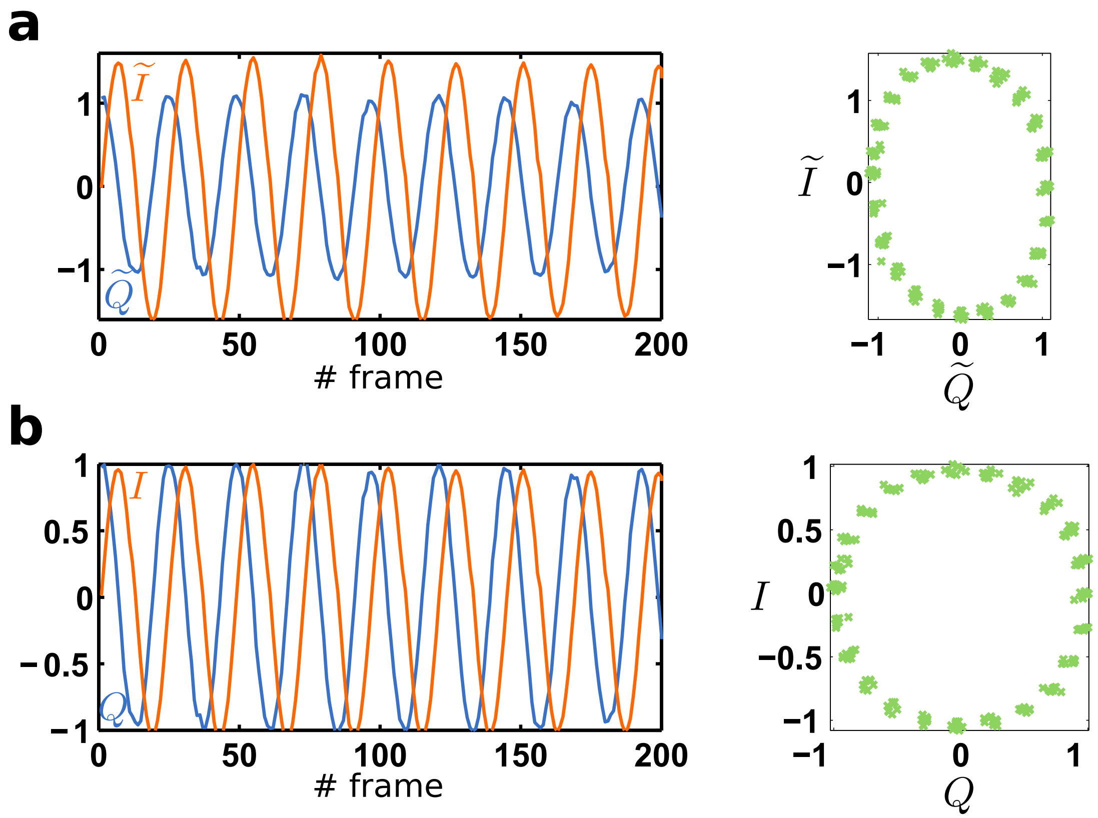

Supplementary Figure 2: Illustration of the quadrature mismatch correction algorithm. **a** Original quadrature signals ($\tilde{I}$ in red, $\tilde{Q}$ in blue) of the reference pixel (marked with symbol × in Figure 5.b of the main article) as acquired by the camera over 200 frames (350 ms exposure time, 400 ms sampling period) while EO crystal voltage is slowly varied (0.1 Hz). The I-Q representation (green dots) shows clear amplitude mismatch, and slight deviation from 90° phase (quadrature) between $\tilde{I}$ and $\tilde{Q}$ signals. **b** I and Q quadrature signals after correction showing equal amplitudes and perfect quadrature angle.

The experimental data in this case can be modeled by writing the two quadrature transmission functions at each pixel $(i,\ j)$ of the FOV as

$$\begin{aligned} T_{\tilde{I}_{(i,\ j)}} &= T_{I_1,(i,\ j)} - T_{I_2,(i,\ j)} = \cos(2\pi f_{\mathrm{d}} t + \phi_{(i,\ j)}), \text{ and} \\ T_{\tilde{Q}_{(i,\ j)}} &= T_{Q_1,(i,\ j)} - T_{Q_2,(i,\ j)} = \alpha_{(i,\ j)} \sin(2\pi f_{\mathrm{d}} t + \phi_{(i,\ j)} + \delta\phi_{(i,\ j)}), \end{aligned} \tag{1}$$

where $\alpha_{(i,\ j)}$ accounts for the amplitude mismatch between the two quadratures at pixel $(i,\ j)$, whereas $\delta\phi_{(i,\ j)}$ stands for phase deviation from perfect quadrature between $T_{\tilde{I}_{(i,\ j)}}$ and $T_{\tilde{Q}_{(i,\ j)}}$. It can be noted that a pixel-dependent common phase factor $\phi_{(i,\ j)}$ has been included in the above equation, which models the inhomogeneous phase distribution across the FOV, due to the isogyre pattern. As mentioned in Supplementary Note 1, this phase distribution is compensated in the last step of the frames processing during the phase unwrapping step to provide a correct estimated phase map. As a result, for the sake of clarity in the following description of the quadrature mismatch calibration and correction procedure, we will set $\phi_{(i,\ j)} = 0$ without loss of generality.

The objective of the quadrature mismatch calibration described below is to estimate the amplitude mismatch map α, as well as the phase quadrature mismatch $\delta\phi$ across the FOV, i.e., at each pixel $(i,\ j)$. Several calibration methods exist for correcting the intensity and phase of a quadrature demodulator, especially for their application in RADARs [2, 3]. We estimated the α and $\delta\phi$ maps from calibration, using a time-series image acquisition of $N = 200$ raw frames, such as the one presented here in Supplementary Figure 2.a for the reference pixel marked with a red cross symbol in Figure 5.b of the main article. The $N = 200$ images temporally sample the amplitude response of the two quadrature images $\tilde{I}$ and $\tilde{Q}$ over approximately 8 periods, with a spatially homogeneous constant (unmodulated) illumination on the FAST-QUAD prototype, while the voltage applied on the EO crystal was slowly varied ($f_\mathrm{d} = 0.1$ Hz). Indeed, at a given pixel $(i,\ j)$ of the image, the transmission can be written as a $N \times 2$ matrix

$$\mathrm{T}_{(i,\ j)} = \begin{bmatrix} (T_{\tilde{I}_{(i,\ j)}})_1 & (T_{\tilde{Q}_{(i,\ j)}})_1 \\ \vdots & \vdots \\ (T_{\tilde{I}_{(i,\ j)}})_N & (T_{\tilde{Q}_{(i,\ j)}})_N \end{bmatrix}, \tag{2}$$

whose covariance matrix yields

$$\left\langle \mathrm{T}^T_{(i,\ j)} \mathrm{T}_{(i,\ j)} \right\rangle = \begin{bmatrix} 1 & \alpha_{(i,\ j)} \sin \delta\phi_{(i,\ j)} \\ \alpha_{(i,\ j)} \sin \delta\phi_{(i,\ j)} & (\alpha_{(i,\ j)})^2 \end{bmatrix}, \tag{3}$$

showing that $\alpha_{(i,\ j)}$ and $\delta\phi_{(i,\ j)}$ can be easily estimated from the calibration data. It can be noted that in the ideal case, the covariance matrix should be equal to the identity matrix, denoting perfect amplitude balancing and exact quadrature phase.

Thus, one can estimate a scaling and a rotation matrix at each pixel $(i,\ j)$ that can be applied to the observed data $\mathbf{X}_{(i,\ j)} = [\tilde{I}_{(i,\ j)}\ \tilde{Q}_{(i,\ j)}]$ to balance the amplitudes and obtain perfect quadrature signals. To calibrate these correction matrices at each pixel, we follow a well-known method based on singular values decomposition (SVD) and similar to [3]. Briefly, the SVD of matrix $\mathrm{T}_{(i,\ j)}$ is computed as $\mathrm{T}_{(i,\ j)} = A_{(i,\ j)}\Sigma_{(i,\ j)}B^T_{(i,\ j)}$, and one can easily extract the 2×2 real unitary matrix $B^T_{(i,\ j)}$, as well as the two first singular values stored in the 2×2 diagonal matrix $\Sigma'_{(i,\ j)}$ (which is the 2×2 sub-matrix of the $N \times 2$ matrix $\Sigma_{(i,\ j)}$). As shown in [3], it suffices to apply a correction matrix $R_{(i,\ j)} = B_{(i,\ j)}\Sigma'^{-1}_{(i,\ j)}B^T_{(i,\ j)}$ to the observed quadrature data $\mathbf{X}_{(i,\ j)}$ to obtain corrected data $\mathbf{X}_{(i,\ j)}R_{(i,\ j)}$. The efficiency of this correction algorithm can be checked in Supplementary Figure 2.b where the corrected quadratures I and Q are displayed, showing equalized amplitude and perfect quadrature phase.

SUPPLEMENTARY NOTE 3: DESCRIPTION OF THE SCENES BEING IMAGED

The scenes that were being imaged were illuminated with a 532 nm (Coherent Verdi) green laser. Acousto-optic modulators (AOM) (AA Opto-Electronic, MT80-A1-VIS) were used to obtain intensity-modulated beams. Indeed, by supplying the AOMs with RF signals whose amplitude was modulated at frequency f, the light diffracted in the first diffraction order shows an intensity modulation at frequency f with modulation index $m \simeq 100$ %. A sketch of the illumination setup is given in Supplementary Figure 3 below. Direct modulation of the LEDs or laser light sources can be preferentially envisaged in future developments and applications of FAST-QUAD. To obtain an image of an homogeneously illuminated scene, the modulated beam was directed into an 8 inches diameter integrating sphere (Labsphere CSTM-US-800C-100R) which created a uniform illumination field across its main circular aperture of 5 cm diameter. Note that during the experiments the sphere was mechanically connected to an electrodynamic shaker (with frequency far from the demodulation frequency, typ. ~ 100 Hz) which created small vibrations of the sphere, thereby limiting the detrimental effect of speckle on the acquired images. To produce the images displayed in Figure 2 of the main article, a 4 cm mask of the logo of the Institut Foton was printed on a transparency film and positioned in front of the aperture of the integrating sphere. For the last experiments presented in Figure 4 of the main article, the two collimated laser spots (modulated at distinct frequencies with two AOMs) directly illuminated a rotating white diffuser (paper) used in place of the integrating sphere to avoid speckle (Figure 4.a). In Figure 4.b, the collimated laser beams were used to illuminate white-dotted images of the lock (unmodulated) and key (modulation frequency 5 kHz), both printed on a transparency film. Two lenses were finally used to superimpose the final images of the lock and key on the rotating diffuser and create the scene of Figure 4.b. This optical projection arrangement was subject to some geometrical aberrations in the projection lenses, hence resulting in apparent lower resolution of the images in Figure 4.b of the main article.

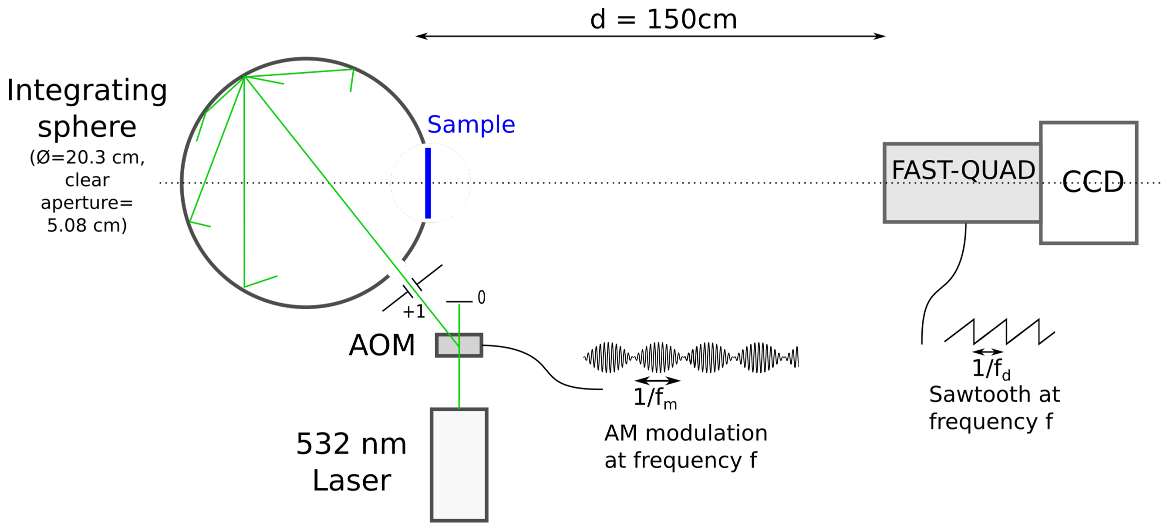

Supplementary Figure 3: Schematic of the experimental setup for imaging experiments with the FAST-QUAD prototype.

SUPPLEMENTARY REFERENCES

[1] Fade, J. et al. Long-range polarimetric imaging through fog. *Appl. Opt.* **53**, 3854–3865 (2014).

[2] Churchill, F. E., Ogar, G. W. & Thompson, B. J. The correction of I and Q errors in a coherent processor *IEEE T. Aero. Elec. Sys.* **17**, 131–137 (1981).

[3] Noon, D. A., Longstaff, I. D. & Stickley, G. F. Wideband quadrature error correction (using SVD) for stepped-frequency radar receivers. *IEEE T. Aero. Elec. Sys.* **35**, 1444–1449 (1999).
